# Personalized tremor control targeting for MR-guided focused ultrasound using tractography

**DOI:** 10.1093/braincomms/fcag170

**Published:** 2026-06-22

**Authors:** Noam Shalem, Alon Sinai, Gil Zur, Haim Azhari, Gal Carmely, Maria Nassar, Inna Senderova, Goni Merhav, Ayelet Eran, Ilana Schlesinger, Lior Lev-Tov

**Affiliations:** Faculty of Biomedical Engineering, Technion Israel Institute of Technology, Haifa 3200003, Israel; Department of Neurosurgery Rambam Health Care Campus, Haifa 3525408, Israel; Department of Neurosurgery Rambam Health Care Campus, Haifa 3525408, Israel; Department of Radiology Rambam Health Care Campus, Haifa 3525408, Israel; Faculty of Biomedical Engineering, Technion Israel Institute of Technology, Haifa 3200003, Israel; Faculty of Biomedical Engineering, Technion Israel Institute of Technology, Haifa 3200003, Israel; Department of Neurology Rambam Health Care Campus, Haifa 3525408, Israel; Department of Neurology Rambam Health Care Campus, Haifa 3525408, Israel; Department of Radiology Rambam Health Care Campus, Haifa 3525408, Israel; Department of Radiology Rambam Health Care Campus, Haifa 3525408, Israel; Department of Neurology Rambam Health Care Campus, Haifa 3525408, Israel; Faculty of Medicine, Technion Israel Institute of Technology, Haifa 3200003, Israel; Faculty of Biomedical Engineering, Technion Israel Institute of Technology, Haifa 3200003, Israel; Department of Neurosurgery Rambam Health Care Campus, Haifa 3525408, Israel; Faculty of Medicine, Technion Israel Institute of Technology, Haifa 3200003, Israel

**Keywords:** personal targeting, modelling DRT, tracts normalization, personalized neuromodulation, tractography validation

## Abstract

Magnetic Resonance guided Focused Ultrasound Surgery (MRgFUS) is a non-invasive, innovative technology that induces a thermal lesion to treat tremor in medication-resistant patients diagnosed with essential tremor or Parkinson's disease. The ventral intermediate nucleus of the thalamus (VIM) is a well-established neuromodulation target for tremor reduction. The standard VIM detection method is based on anatomical landmarks, specifically the anterior and posterior commissure. However, it is correlated with suboptimal tremor control up to 20%, inconsistency of the treatment's outcome, leading to longer procedures with excessive amounts of sonications and adverse events. The use of tractography as a targeting tool is gaining momentum. However, validation and implementation are needed to establish tractography as a superior VIM targeting method. This study suggests a novel personalized targeting approach for treatment, leading to superior clinical outcomes. Pre- and postoperative magnetic resonance imaging scans were obtained from 81 patients who underwent MRgFUS between 2014 and 2021. Using preoperative diffusion tensor imaging, three tracts (pyramidal tract, medial lemniscus, and dentatorubrothalamic tract [DRT]) were identified for each patient using tractography. The postoperative T1 weighted images were used for segmentation, volumetric lesion evaluation, and correlation to the tracts. Clinical outcome was evaluated up to 2 years postoperatively and correlated to each patient's lesion characterization. Retrospective analysis of lesion location relative to a personalized DRT-based coordinate system has shown that successful lesions are consistently located in the anterolateral region of the DRT, whereas failed-treatment lesions encircle them. A linear regression model using tract features predicted optimal target locations with high accuracy (*R*^2^ = 0.84). In addition, a specific circular region of the tract in which at least 85% of the success lesions overlap was detected, while a ‘critical area’ of maximum success-fail difference was observed in its posterior-lateral margins. Using normalized ‘sweet spot’ DRT coordinates can distinguish successful from failed lesions with a *P*-value < 0.0001. Other factors, such as lesion volume and area at the anterior and posterior commissure plane, lesioned DRT volume and area, and anatomical coordinates, showed no correlation with treatment outcome, underscoring the importance of lesioning the specific part of the DRT for successful long-term tremor control. These results reveal an innovative personalized tool for optimal targeting of tremor control treatment, while further investigation should examine the targeting approach outcome superiority.

## Teaser

Shalem *et al*. report a novel personalized tractography-based targeting method for tremor reduction using MR guided focused ultrasound. They leverage tract centre-of-mass metrics, dentatorubrothalamic tract modelling, and lesion analysis to identify optimal targets, which highlight a specific subregion within the dentatorubrothalamic tract that is correlated with superior outcome: tremor control and quality-of-life improvement.

## Graphical Abstract

**Graphical Abstract:**
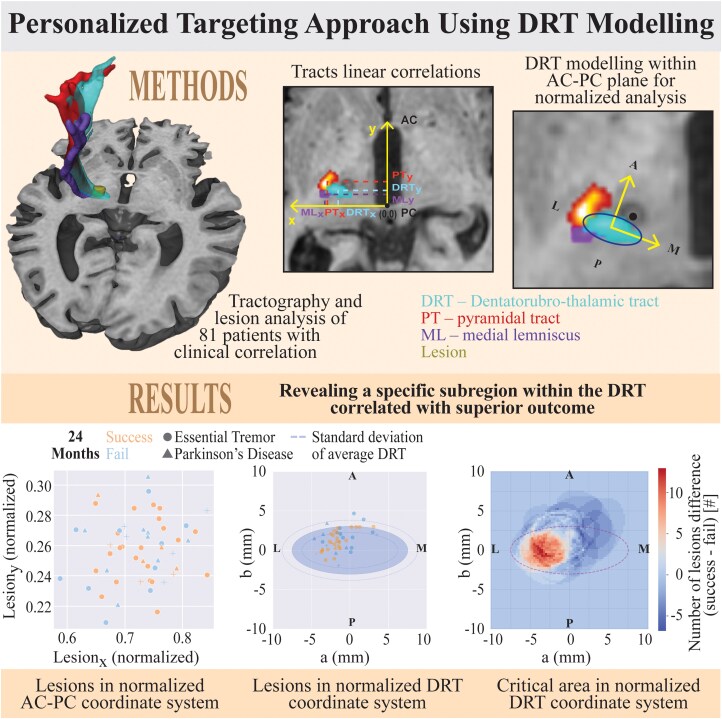

## Introduction

Tremor is a symptom of both Essential tremor (ET) and Parkinson's disease (PD), which can dramatically reduce the quality of life and performance of daily routine tasks.^1,2^ Surgical treatments such as Deep Brain Stimulation (DBS), radiofrequency (RF) thalamotomy, and radiation (Gamma knife) thalamotomy are available for medication-resistant patients.^3^ Magnetic Resonance guided Focused Ultrasound Surgery (MRgFUS) is a non-invasive technology utilizing ultrasound to induce a focal thermal lesion with sub-millimetre precision to treat patients with tremor.^3-5^ Small focal diameter (2–4 mm), submillimetre steering capability, and real-time neurologic and imaging monitoring, enabling targeting optimization with respect to tremor control and minimizing SE.

The anatomical target location is the most important single factor for clinical outcome and tremor control. The ventral intermediate nucleus of the thalamus (VIM) is the preferred target for ET and tremor-dominant PD patients.^3,5-9^ The VIM, which is involved in the neurofunctional circuits of motion control, occupies ∼0.5–2.0% of the total thalamic volume.^10-14^ However, even with high-resolution MRI, VIM detection is limited by insufficient intrinsic contrast in the thalamus.^10,15^ Furthermore, the specific location of tremor control within the VIM remains unsubstantiated, whereas VIM lesioning or stimulation is associated with motor and sensory side effects (SE), including dysarthria and disequilibrium.^7^ The standard method for VIM detection is based on anatomical landmarks, specifically the anterior commissure (AC) and posterior commissure (PC). This indirect targeting method is based on histological atlases,^16^ not patient-specific and, as a result, insensitive to anatomical variances,^10,17-20^ resulted in heterogeneity in the coordinates used across medical centres.^10,14^ In contrast, the growing field of direct targeting approaches aims to tailor the target location directly from the patient’s MRI, however, none of these approaches have demonstrated sufficient accuracy, reliability, or superior outcomes.^5,10,21-25^ Nonetheless, studies suggest that MR diffusion tensor imaging (DTI) with tractography of specific tracts is an emerging technique for direct targeting of the VIM.^22,26^

Tractography is the only non-invasive tool for identifying white matter pathways in vivo.^27^ Although it suffers from several limitations, such as poor resolution and difficulty resolving complex intra-voxel fibre crossings or non-dominant fibre populations, its non-invasive nature makes it an indispensable tool for addressing multiple scientific and clinical needs.^16,27-29^

The use of tractography as a clinical and research tool is increasing and shows promise for targeting and improving neuromodulation outcomes.^26,30-32^ Various studies employing diverse tractography methods have been published in recent years, describing correlations between prospective or retrospective tractography for DBS and MRgFUS and treatment outcome and SE. However, the vast majority of these reports include small cohorts (≤20) and provide up to 1 year of postoperative clinical follow-up.^10,25,26,32-36^ To date, there is no established method for tractography-based targeting for tremor reduction using MRgFUS. Further research is needed to validate and implement tractography as the primary method for localizing the VIM to improve patient outcomes.

Three main tracts identified by tractography provide a means of localizing the VIM. The borders of the VIM are detected by the corticospinal tract (CST) and medial lemniscus (ML), while the dentatorubrothalamic tract (DRT) connects the cerebellum with the cerebral cortex, traversing the VIM.^26,30,37^ Various studies emphasize the importance of directing the DRT in DBS and MRgFUS procedures. Moreover, evidence shows that abnormal activity in the DRT is part of the pathophysiology of ET^16,34^ while disrupting the DRT is essential for clinically effective VIM lesioning with MRgFUS.^8,26,34^ In DBS, electrodes localized within or near the DRT are associated with superior outcomes.^29,34^

MRgFUS lesioning, using the AC-PC targeting method, is correlated with suboptimal tremor control up to 20%, especially regarding the long-term effect and SE.^7^ Inaccuracy targeting results in a longer procedure with excessive sonications, which may affect treatment outcomes, adverse events, and patient cooperation. There is an unmet need to optimize lesioning and modulation targeting within the VIM to improve tremor control and durability, minimize SE, and enhance patient compliance and tolerance by reducing treatment duration, number of sonications, and energy. Hence, we used retrospective data based on 10 years of MRgFUS treatments at our centre. Utilizing personal tractography and lesion analysis to identify parameters contributing to long-lasting tremor reduction and establish a new targeting approach for tremor control and superior clinical outcomes.

## Materials and methods

### Study cohort

The cohort of this study includes 81 patients between the ages 45–87 (69.15 ± 7.48), both female and male (23, 58, respectively), who underwent MRgFUS for tremor reduction and were diagnosed at the time of treatment with ET or tremor-dominant PD (57, 24, respectively).

Clinical outcome was evaluated using the Unified Parkinson's Disease Rating Scale (UPDRS) Part III for PD patients and the Clinical Rating Scale for Tremor (CRST) questionnaire for ET patients. Because Parkinson’s patients may present with other motor (and non-motor) symptoms, whereas VIM thalamotomy primarily reduces tremor, a general tremor score [TS] and a tremor score of the treated hand [TS(th)] were calculated. Using the UPDRS tremor subcomponents (issues 20–26 with a maximum score of 28) of the treated side, which was reported elsewhere,^38^ and the CRST tremor subcomponents of the treated side allows us to focus on the tremor reduction effect regardless of the patient’s diagnosis and evaluate tremor control in a personalized fashion with respect to baseline. In addition, a quality of life (QOL) assessment was performed using the PD questionnaire (PDQ-39) for PD patients and QOL in essential tremor (QUEST) questionnaire for the rest of the patients. The local Helsinki Committee (Institutional Review Board) approved the study.

### MRI image acquisition

Whole-brain structural and diffusion-tensor MRI images were acquired before and after the procedure on a 3.0-T system (Discovery MR750; GE Healthcare, Milwaukee, Wis): three-dimensional spoiled gradient-recalled-echo axial T1-weighted images (repetition time msec/echo time msec, 8.3/3.23; 0.86 × 0.86 × 0.6 mm^3^ voxel dimensions), diffusion-tensor imaging (9538/82; Δ/δ, 33/26; *b* value, 1000 s/mm^2^; 25 or 60 directions of diffusion gradients and five *b*_0_; 2 × 2 × 2 mm^3^ voxel dimensions). Since the DTI protocol was improved over the years, the scans of 15 patients were acquired with 60 directions of diffusion gradients, compared with 25 for the rest of the patients (66 patients).

### Clinical data processing

Clinical scores were consistently noted at movement disorder specialist follow-up visits. TS and TS(th) were extracted along with QOL at the preoperative visit and used as the baseline; postoperative tremor scores were normalized to baseline to present changes as a percentage for each patient.

Clinical scores were evaluated at 3, 6, 12, 24, and 36-month follow-ups. Success criteria were calculated based on TS(th) ≤ 0.4 (i.e. tremor reduction ≥ 60% in the treated-hand side) and QOL at 6 months < 1 (i.e. there was a clear improvement in QOL at 6 months with respect to baseline) for each follow-up time-point.

The 6-month time point was chosen for QOL to eliminate transient side effects (likely secondary to perilesional oedema) and to prolong deterioration in QOL attributable to the pathology's natural history. Once treatment was determined to be a failure (i.e. not success) at some point, all subsequent follow-ups were scored as failures regardless of the clinical scores.

### Tractography processing

Each patient's pre-operative DTI and T1 scans were processed for tract analysis using the software ExploreDTI.^39^ Tensors were calculated using a robust estimation algorithm, corrected for motion distortions and eddy currents.

**Figure 1:**
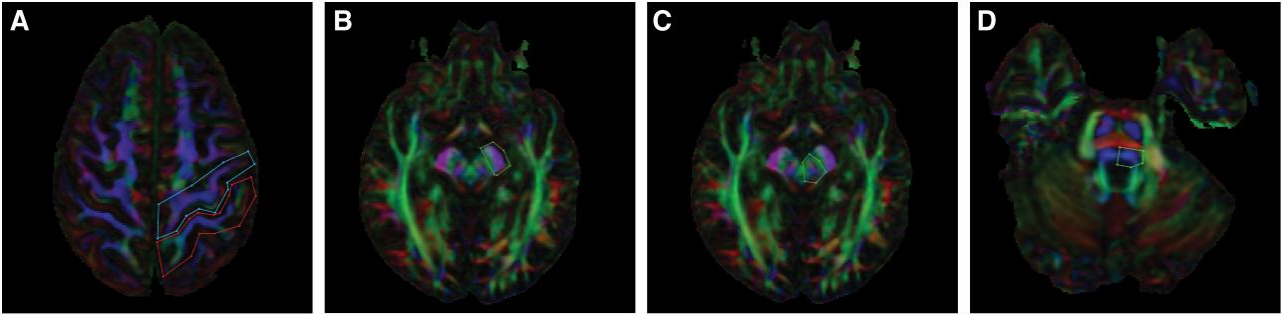
**The ROIs locations for the tracking process of the PT, ML, and the DRT on FA colour map.** Over the (**A**) primary motor cortex (blue) for tracking the PT and the DRT, primary sensory cortex for tracking the ML (red), (**B**) cerebral peduncle for tracking the PT, (**C**) red nucleus for tracking the DRT, and (**D**) dorsal column at the brain stem for tracking the ML. DRT, dentatorubrothalamic tract; FA, fractional anisotropy; ML, medial lemniscus; PT, pyramidal tract; ROI, region of interest.

Whole-brain fibre orientation distribution–based tractography was performed by using the constrained spherical deconvolution method (fibre orientation distribution threshold, 0.1; 60° maximum deviation; 1-mm steps, and fibre length was 50–350 mm).^39^ The most relevant tracts targeting the VIM are the DRT, ML, and PT, which help to identify the patient-specific functional anatomy of the VIM, the tremor pathway, and its surroundings. For each tract, regions of interest (ROIs) were marked using the fractional anisotropy map for tracing (Fig. 1), detailed methodology is provided in the supplementary. All the tracts were reviewed for quality assessment to validate anatomical characterization and ensure uniform cleaning. Tracts that were highly sparse, had exceptionally low volume, or were extremely diverse from typical tract morphology were classified as poor quality and eliminated. In addition, patients with two or more tracts of significantly poor quality were excluded from the study.

For each patient, all postoperative T1 scans, the pre-operative DTI scan, and the tracts were registered to the pre-operative T1 scan. This process includes stripping the skull for the b_0_-DTI and T1 scans with the ‘BET’ algorithm^40^ (by FSL)^41-43^ and the ‘antsBrainExtraction’ algorithm [by Advanced Normalization Tools^44^ (ANTs)], respectively.^45^ Brain extraction was performed for improved registration, however, in some cases, accurate alignment was obtained only with the skull. The registration was performed by the ‘antsRegistrationSyN’ algorithm (by ANTs),^44^ executing an affine transformation. All registrations were manually reviewed by a single rater to ensure sufficient accuracy, and inaccurate registrations were eliminated from the study.

### Lesion analysis and geometrical measurements

In this study, the follow-up protocol included full MRI scans, hence, lesion imaging evaluation over time was feasible. However, lesion analysis was based on one-day postoperative (POD1) T1 MRI scans, as these scans most closely resemble the planned lesion's centre, area, and volume, while peri-lesional oedema can be easily distinguished from the lesion without significant impact. Lastly, it can be reliably registered to the pre-op MRI DTI and projected on the tracts. T1 was chosen due to high *z*-axis resolution (0.6 mm slice thickness) and the lesion core can be easily identified, increasing the lesion analysis accuracy.

For the lesion analysis and all geometrical measurements, self-written MATLAB™ scripts were used. The segmented lesion was manually examined by a single rater to ensure reproducibility, and segmentation adjustments were made as needed.

Density images were generated for each tract, while the value of a voxel was determined by the number of fibres passing through it. Since the *z*-axis resolution of the T1 scan (0.6 mm) is higher than in the DTI scan (2 mm), when registering a tract to the T1 space, a slight dilation occurs due to the interpolation. Hence, to create a binarized mask of each tract while minimizing the effects of the registration on the tracts, the values were rescaled to 0–100, and pixels with values above one were considered part of the tract.^46,47^ This method showed the best resemblance between the original and registered tracts.

**Figure 2:**
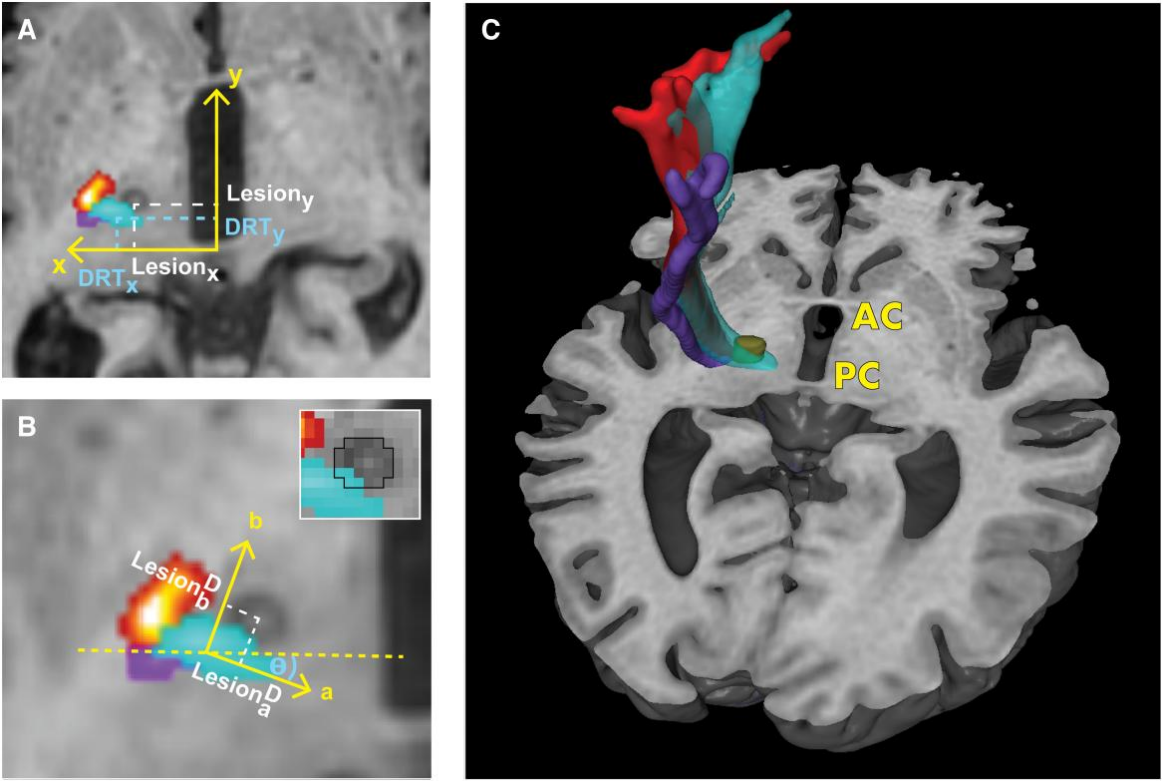
**Lesion evaluation with respect to the DRT (cyan), PT (red), and ML (purple) using two coordinate systems.** (**A**) AC-PC coordinate system, origin at the PC point. Positive × toward the treated thalamus. (**B**) DRT coordinate system normalized to the ellipse long axis (rotated by Ѳ). Origin at the DRT COM. Positive a toward the midline. Note, lesion geometry analysis at T1 MRI (**B**). (**C**) 3D visualization of the three tracts and the lesion (dark yellow). Lesion^D^, Lesion’s COM coordinates with respect to the DRT coordinate system. AC, anterior commissure; DRT, dentatorubrothalamic tract; ML, medial lemniscus; PC, posterior commissure; PT, pyramidal tract.

Subsequently, we calculated the lesion's volume and 3D positioning within the T1 space concerning the surrounding anatomical markers and the analyzed tracts (Fig. 2C). Specific parameters were collected, such as the area under the AC-PC plane, the distance between the lesion's centre of mass (COM) to the AC-PC in three axes, and the percentage of overlap between the lesion and each tract volume. A special investigation was performed at the ACPC level axial plane: the lesion overlap area with DRT, distances between the tracts’ COMs and the lesion's COM, and the distance between the lesion's COM (final target) and the preliminary planned target (which was calculated canonically 14 mm from midline, 0.25×ACPC at the ACPC plane).

For further analysis and measurements, a coordinate system was considered, such that the origin is the intersection between the third ventricle midline and the PC, where the midline is the *y*-axis, and the positive direction of the *x*-axis is toward the treated side (Fig. 2). The lesion and tracts’ COMs locations with respect to the *X* and *Y* axes are presented as $\mathrm{trac}t_{X},\;\mathrm{trac}t_{Y}$. The AC-PC length was measured manually with pixel size resolution (0.86 mm).

Evaluating DRT at the ACPC level revealed mediolateral ‘smearing’; hence, we modelled the DRT as an ellipse that can be defined by its two diameters (D1, D2), orientation, and COM, which coincide with the tract's COM (Fig. 2). This approach enables a practical way for tract normalization and comparison between patients.

To examine the relationship between treatment outcomes and lesion locations relative to the DRT, a second coordinate system was defined with respect to the DRT ellipse model. The origin is the ellipse COM, while ‘a’ and ‘b’ corresponds to the long and short ellipse axes, respectively, rotated by angle Ѳ_DRT_ with respect to the X-Y coordinate system (Fig. 2). In this manner, the coordinate system is fitted to each patient's DRT, and the relative location of the patient's lesion on its DRT (on axis ‘a’ and ‘b’) will be denoted as ${\mathrm{Lesion}}_{a}^{D},\;\;{\mathrm{Lesion}}_{b}^{D}$ (Fig. 2B).

### Statistical analysis

A further linear regression analysis examined the correlation between the tracts and the treatment's outcome. According to the criteria described, a linear regression model was built based on data from patients whose treatment was classified as successful at 24 months. The same model was evaluated utilizing a leave-one-out method and applied to the group of patients whose treatment was classified as failed at 24 months. The r-squared, the average, and the standard deviation of the Euclidean distance between the predicted and actual targets were calculated to evaluate the model's performance in all three cases.

## Results

### Clinical evaluation

During the years 2014 to 2021, 121 patients underwent unilateral MRgFUS thalamotomy at Rambam Medical Centre, while 81 patients were included in the analysis (with appropriate imaging and follow-up documentation). At 1 year follow-up analysis 46 were classified as success treatment with mean 0.11 ± 0.11 TS(th) (i.e. tremor reduction of 89%), and 0.28 ± 0.23 QoL score (72% improvement in QoL). 25 patients were classified as failure treatment, three due to QoL and 22 due to unsatisfying tremor reduction.

At 2 years follow-up 61 patients were included. 34 patients were classified as success treatment with mean 0.14 ± 0.12 TS(th) (86% of tremor reduction), and 0.3 ± 0.25 QoL score (70% improvement in QoL). 27 patients were classified as failure treatment, three due to QoL and 24 due to unsatisfying tremor reduction.

### Tractography

Personalized tractography was performed and three tracts were reconstructed: PT, ML, and DRT. An inter-patient consistency of each tract's trajectory was observed, and the general morphology of the tracts and anatomical landmarks were preserved. At the ACPC level, the PT is located in the internal capsule, lateral to the DRT, and anterior to the ML. The ML and DRT lateral margins adjoin the medial border of the internal capsule, while the ML is located posteriorly to the DRT in the thalamus. The mean locations of the tracts in the AC-PC coordinate system: PT (19.93 ± 1.7 mm, 8.74 ± 1.69 mm), ML (19.22 ± 2.23 mm, 4.43 ± 1.44 mm), DRT (13.90 ± 1.94 mm, 4.89 ± 1.25 mm). The mean area of the DRT at the ACPC plane is 62.68 ± 18.39 mm^2^ (25 diffusion directions: 60.50 ± 16.79 mm^2^, 60 diffusion directions: 70.73 ± 21.52 mm^2^), while the area of the PT is 45.90 ± 12.89 mm^2^, and ML is 19.70 ± 8.42 mm^2^. The quality of the majority of the tracts was satisfactory, while according to coarse quality-based classification: 139 tracts are of good quality (52 PT, 51 ML, 36 DRT), 67 decent (18 PT, 18 ML, 31 DRT), and 33 poor (9 PT, 11 ML, 13 DRT). Out of the poor-quality group four PT, seven ML, and eight DRT were eliminated from the study.

**Figure 3:**
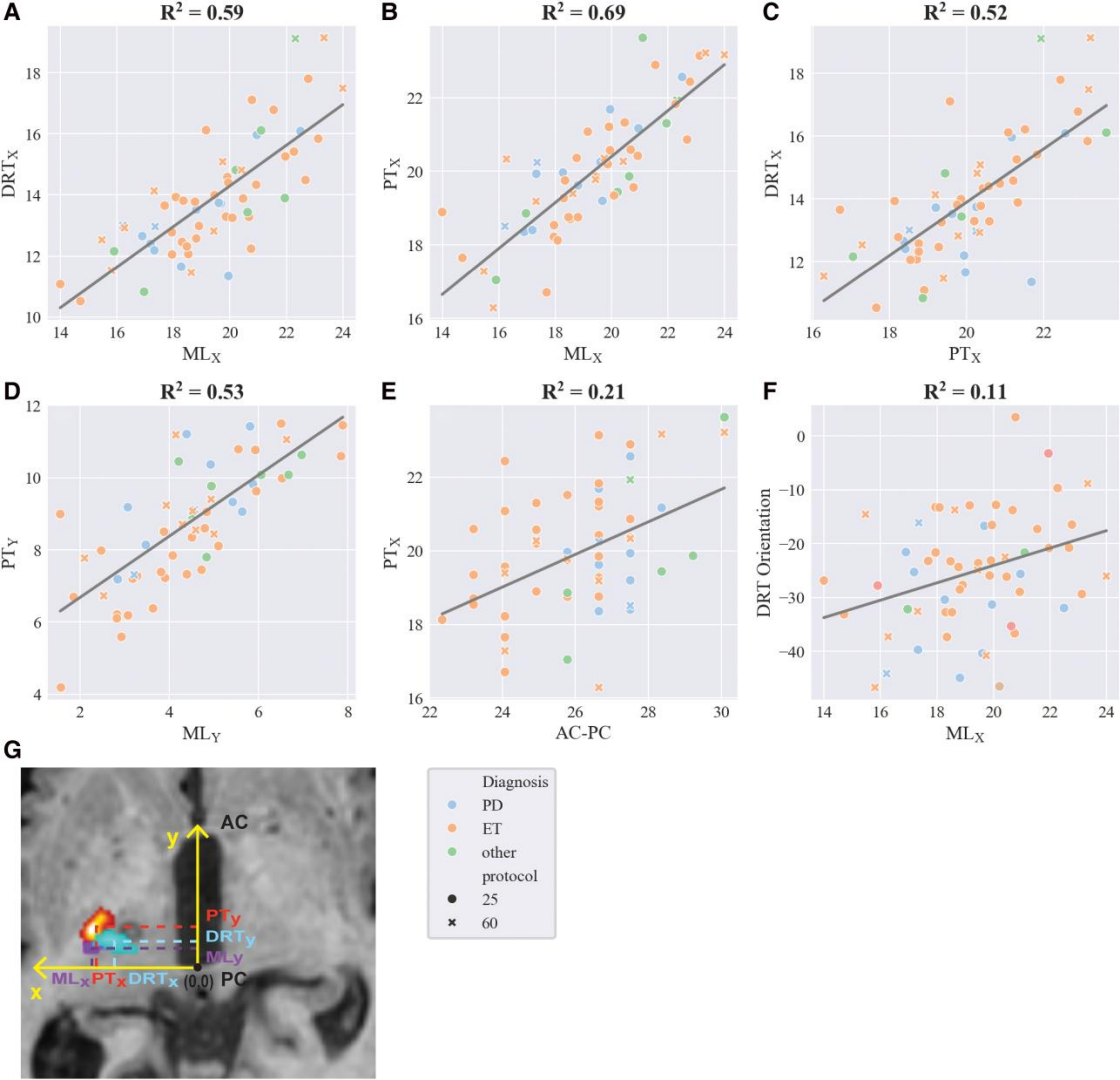
**Tracts correlations.** (**A**)–(**F**) Linear correlations of the tracts’ COMs coordinates, with *R*^2^, each point represents a patient (*N* = 59, 63, 58, 63, 64, and 59 patients, respectively). (**G**) The AC-PC coordinate system with the origin at the intersection of the midline and the PC, and the tracts’ COMs coordinates. AC, anterior commissure; DRT, dentatorubrothalamic tract; ET, essential tremor; ML, medial lemniscus; PC, posterior commissure; PD, Parkinson’s disease; PT, pyramidal tract.

A linear correlation was found between the COMs’ locations of the three tracts: DRT-ML (0.77, 0.6), DRT-PT (0.73, 0.55), PT-ML (0.83, 0.73), (X, Y respectively Pearson correlation coefficient) (Fig. 3). In addition, weaker linear correlations of the DRT's orientation with the PT_Y_ (−0.4), DRT_X_ (0.4) and ML_X_ (0.33), and the AC-PC length with the PT_X_ (0.46) and PT_Y_ (0.42) were found (Fig. 3). No correlation to clinical diagnosis was observed.

No significant variation was found between tracts obtained from scans with 25 and 60 diffusion directions regarding the correlations between the tracts’ COMs and the area of the DRT at the AC-PC plane. However, during tract revision for quality assessment, more tracts acquired with 25 directions were excluded from the study compared with 60 directions (8/1 tracts, respectively), and more patients were excluded due to registration inaccuracies (10/0 patients, respectively).

### Lesion evaluation

**Figure 4:**
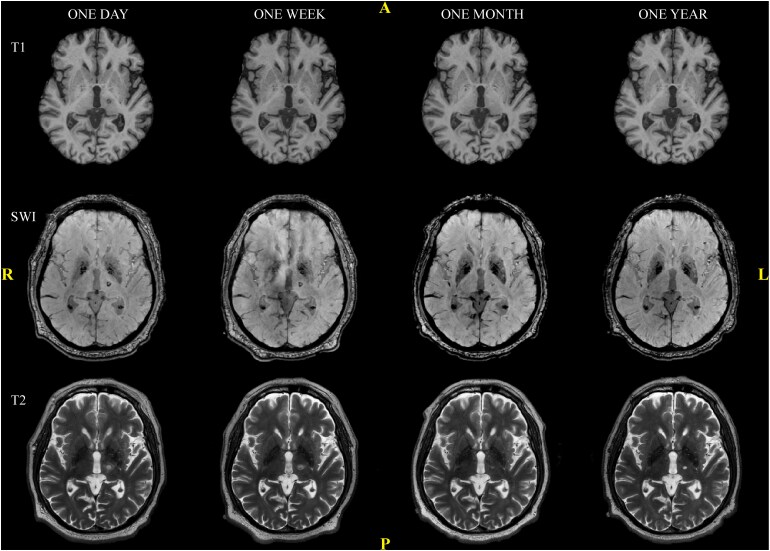
**Lesion evaluation.** Post-operative MRI scans of a patient acquired along follow-up. Rows, top to bottom: T1, SWI, and T2 weighted. Columns, left to right: one day, week, month, and a year after the procedure.

POD1 T1-weighted MRI images were used for lesion evaluation. Lesion size changes throughout imaging follow-up (Postoperative 1 day, 1 week, 1 month, 3 months, 6 months, 1 year). At POD1, the lesion is well-established and may increase at 1 week (while the oedema is most prominent) and decrease incrementally at 1 and 3 months, while it is frequently non-observable on 3-month T1 image (requires SWI or diffusion sequences for evaluation). However, over 6 months, the lesion re-expanded and was detectable (Fig. 4).

**Figure 5:**
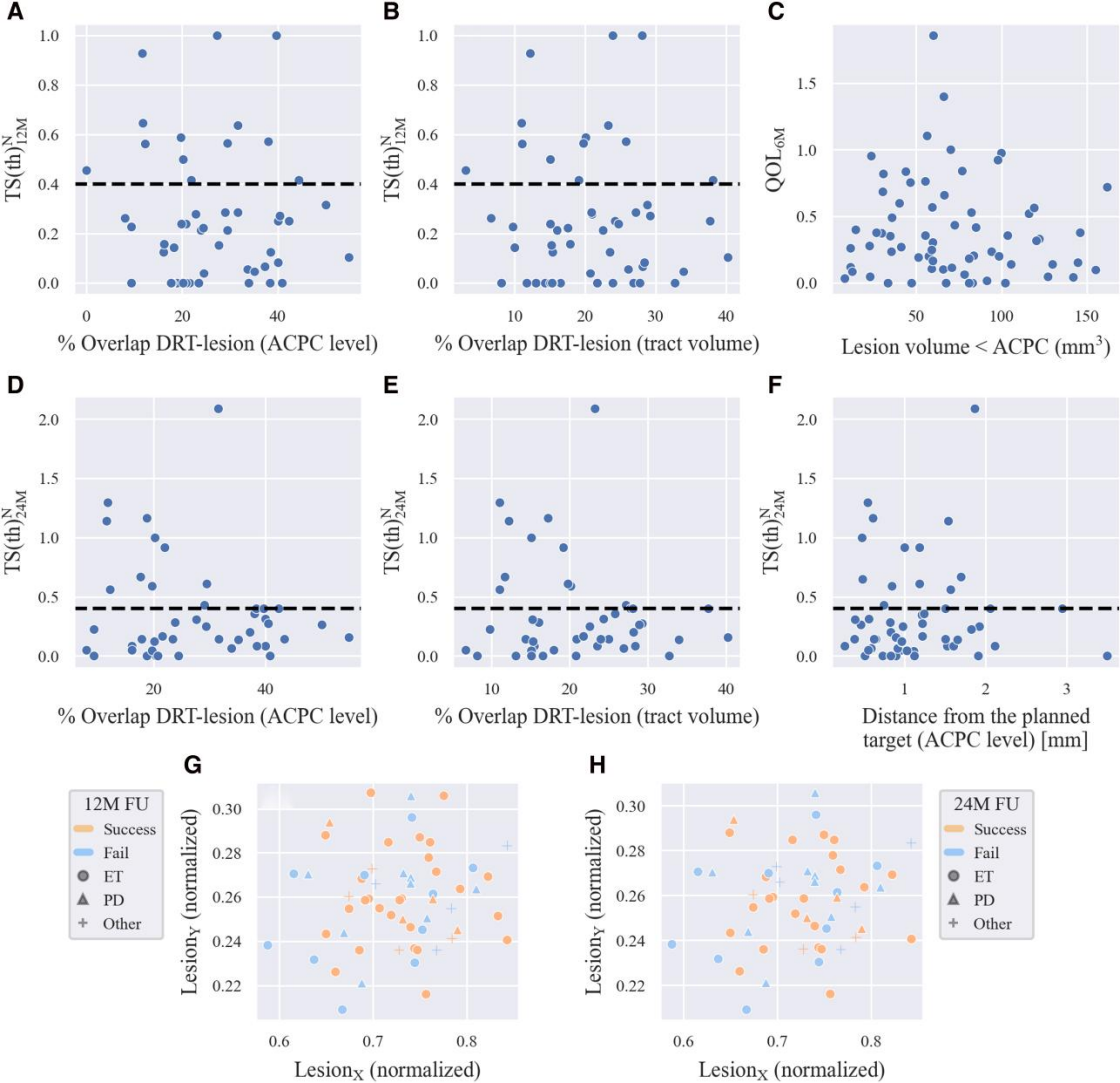
**Lesion spatial evaluation.** Relationship between the percentage lesioned DRT at ACPC plane on POD1 MRI with tremor evaluation at (**A**) 12 months FU (*N* = 51), and (**D**) 24 months FU (*N* = 44), and lesioned DRT volume with tremor evaluation at (**B**) 12 months FU (*N* = 51), and (**E**) 24 months FU (*N* = 44). (**C**) Lesion volume under ACPC plane versus normalized quality of life at 6 months (normalized to preoperative QoL score) (*N* = 66). (**F**) Distance between canonical targeting to the final target versus the tremor evaluation at 24 months FU (*N* = 54). Lesions location COM, at ACPC plane as normalized axes (origin at PC point), clustered treatments at (**G**) 12 months (*N* = 59), and (**H**) 24 months (*N* = 53). Each point represents a patient (*N* = patients). FU, follow-up, TS(th)^N^, normalized to baseline tremor score of treated hand; COM, centre of mass; DRT, dentatorubrothalamic tract; ET, essential tremor; PD, Parkinson’s disease; POD1, postoperative day 1.

**Figure 6:**
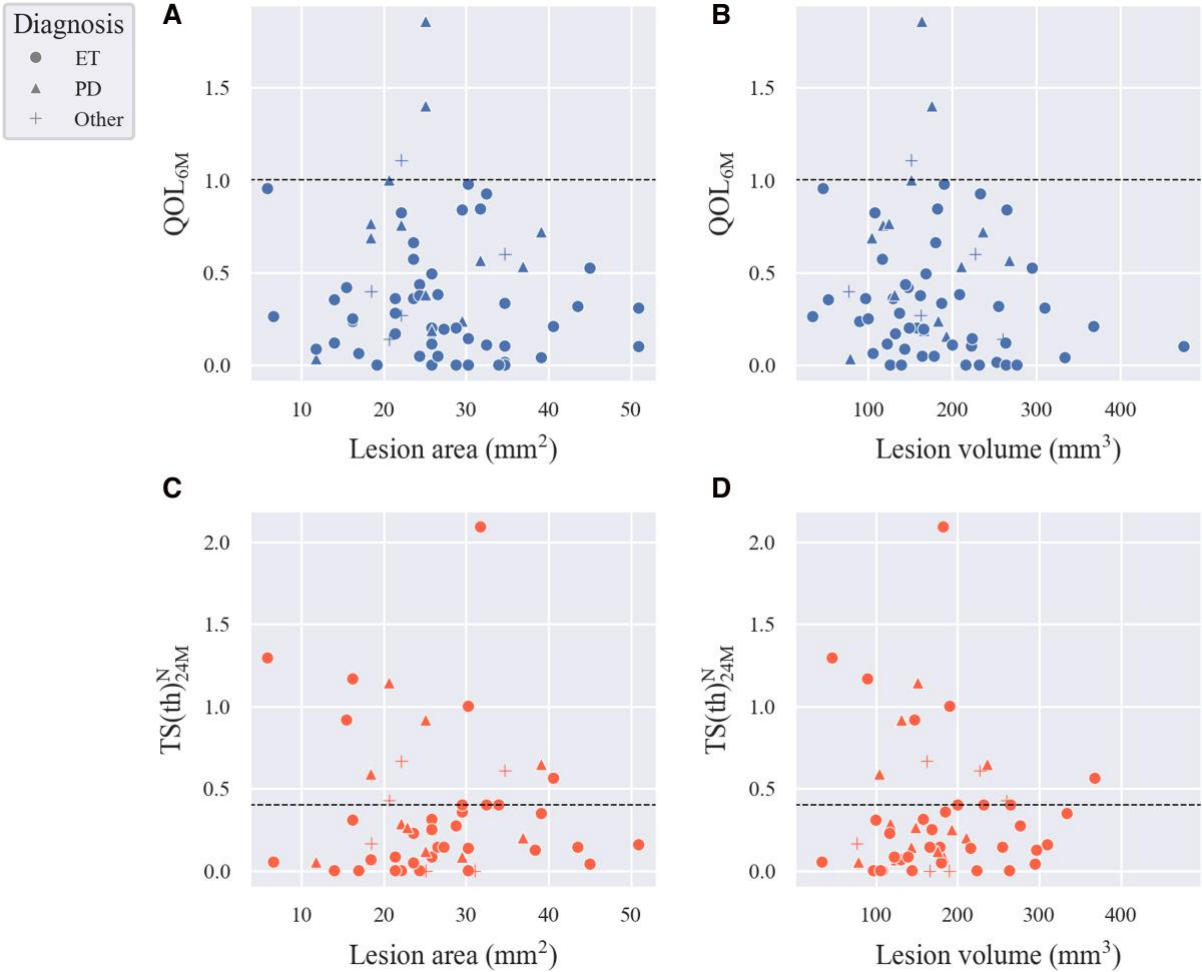
**Lesion area and volume evaluation.** QoL evaluation at 6 months FU (*N* = 66) with respect to lesions (**A**) area at ACPC plane and (**B**) total volume, at POD1 MRI. Tremor evaluation at 24 months FU (*N* = 54) with respect to lesions (**C**) area at ACPC plane and (**D**) total volume, at POD1 MRI. Success-failure threshold as applied in the success criteria (dashed black line). Each point represents a patient (*N* = patients). FU, follow-up, TS(th)^N^, tremor score of treated hand normalized to baseline, COM, centre of mass; ET, essential tremor; PD, Parkinson’s disease; POD1, postoperative day 1.

The lesions geometry parameters (volume, volume under the ACPC plane, and the lesion area at the ACPC plane) were evaluated (Supplementary Table 1), while none of them showed correlation to successful clinical outcome, nor QoL (Figs 5C and 6). The lesion location was evaluated using the COM location at the ACPC plane, the percentage lesioned-DRT volume (at the lesion’s levels) and area at the ACPC plane, and the absolute lesioned-DRT area at the ACPC (Supplementary Table 1). The lesion COM location at the ACPC plane showed no significant correlation to the treatment outcome. Both the percentage lesioned-DRT volume and area at the ACPC plane showed broad-spectrum results without clear correlation to tremor control at 12 and 24 months ${\mathrm{TS}(\mathrm{th})}_{12M}^{N},\;\mathrm{TS}(\mathrm{th}{)}_{24M}^{N}\leq 0.4$ (Fig. 5).

The Euclidean distance between each lesion and the canonical target was calculated, resulting in an average distance of 1.1 mm (±0.61 mm) and no correlation with the TS(th) at 12 and 24 months (Fig. 5F).

A normalized axes system was calculated at the ACPC plane by dividing the *X*-axis (Medial-lateral axis) by the factor PT_X_ (the distance between the PT and the midline) and the *Y*-axis (anterior-posterior axis) by the AC-PC length. Neither the distance from the PC nor the midline showed correlation to superior treatment outcome (Fig. 5G and H).

### Clustering lesions within normalized DRT

**Figure 7:**
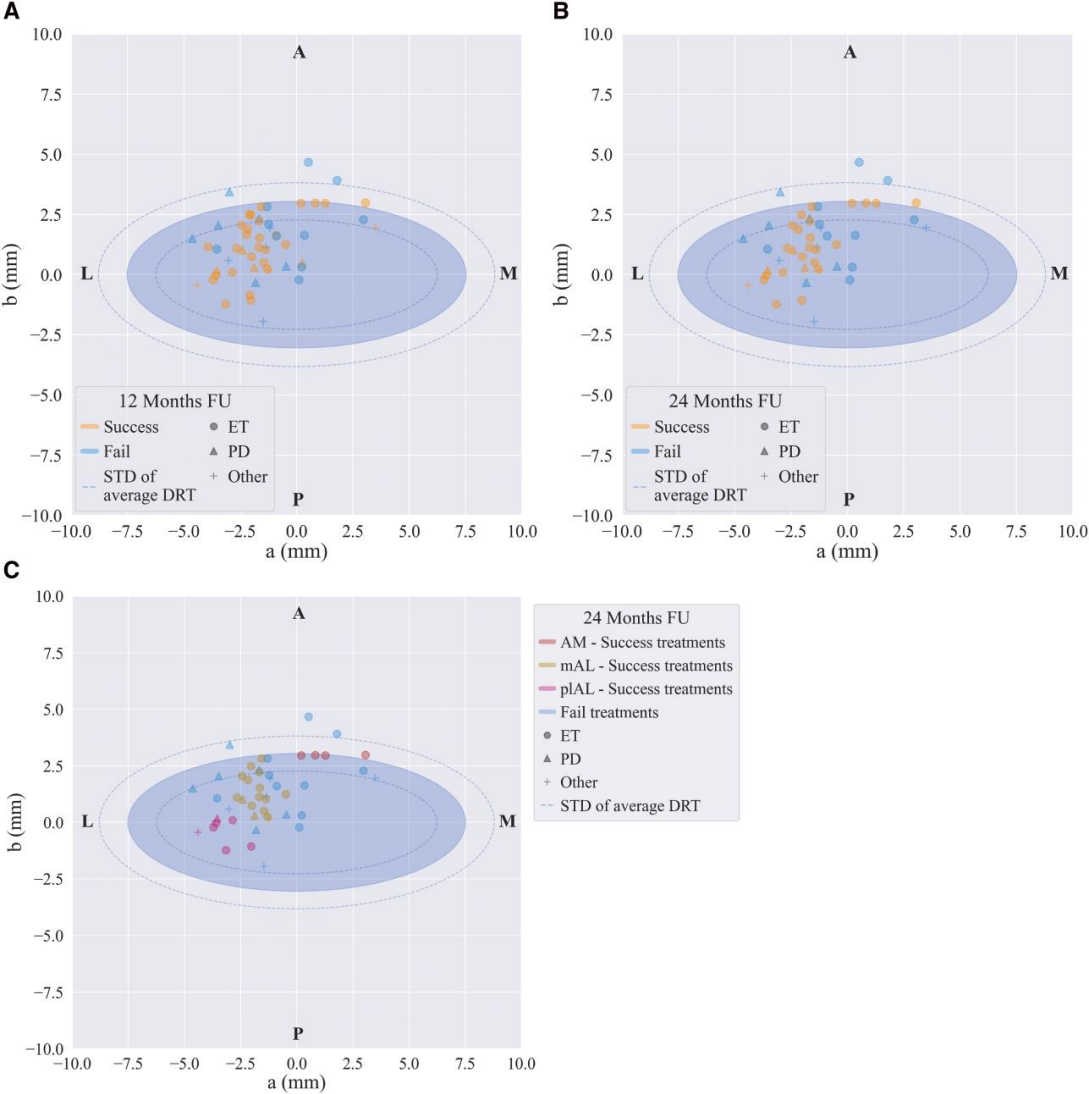
**Lesions COMs-maps.** COMs of all patients’ POD1-lesions in normalized DRT ellipse model (origin at ellipse centre point), coloured as fail and success treatments according to (**A**) 12 months FU (*N* = 54), (**B**) 24 months FU (*N* = 48). (**C**) Clusters of success treatments at 24 months FU. ‘a’, long ellipse axis in [mm]; ‘b’, short ellipse axis [mm], FU, follow-up; COM, centre of mass; POD1, post-operative day 1; DRT, dentatorubrothalamic tract; ET, essential tremor; PD, Parkinson’s disease, STD, standard deviation. A, Anterior; P, Posterior; M, Medial; L, Lateral.

While no significant correlation was noted between canonical targeting and outcome (Fig. 5G and H), we explored lesions COMs with respect to normalized DRT (Fig. 7). Two main clusters were observed. The major one corresponds to the anterior-lateral quarter of the DRT (group AL), while the failure treatments encircle this group. The second successful cluster comprises a small lesion group located at the anterior edge of the anterior-medial quarter of the tract (group AM). This group is explicitly separated from the main AL group in a completely different tract region (Fig. 7C).

The AL group comprises the vast majority of successful lesions and has anterior-posterior smearing. A small cluster of lesions located more posterior-laterally may be separated into a third cluster—the plAL group. The remaining primary condense group is located at the medial part of the anterior-lateral quarter of the DRT–mAL group (Fig. 7C).

Highlighting clusters, the ‘sweet spot’ was defined as the group mean COM location relative to the DRT, and the Euclidean distance from this ‘sweet spot’ was calculated for all successful and failed lesions.

The mAL COMs’ average is −1.75 mm, and 1.29 mm (DRT_a_, DRT_b_ axis, respectively), its mean distance from mAL lesions is 0.88 ± 0.37 mm, and from failure lesions is 2.29 ± 1.34 mm (*P* < 0.0001).

Considering all AL lesions shifts the ‘sweet spot’ postero-lateral to −2.23 mm, 0.78 mm, while its mean distance from the AL COMs is 1.27 ± 0.61 mm, and from failure COMs is 2.55 ± 1.45 mm (*P* < 0.0005).

### Accumulated-overlap map

**Figure 8:**
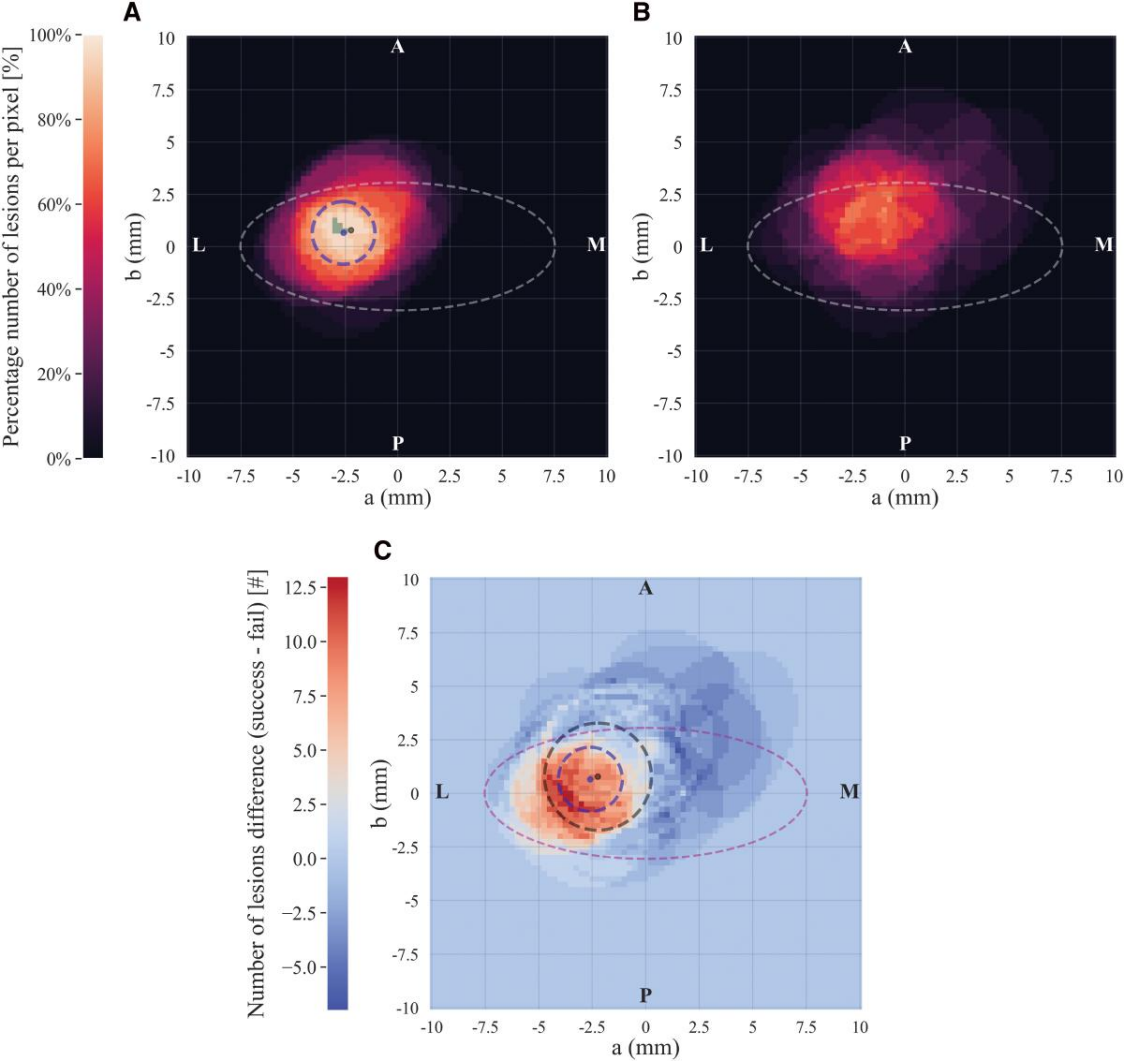
**The accumulated-overlap maps.** All patients’ POD1-lesions accumulated on normalized DRT (grey), divided to (**A**) AL cluster success treatments (*N* = 23) and (**B**) fail treatments (*N* = 21), according to 24 months FU. (**C**) A difference map presenting the subtraction between the AL cluster and fail accumulated-overlap maps on normalized DRT (pink). The target location based on the AL-COMs with 2.5 mm radius (black), the area in which at least 85% of the success lesions overlap with its centre (blue), and the area in which 100% of the success lesions overlap (green). ‘a’, long ellipse axis in [mm]; ‘b’, short ellipse axis [mm]; COM, centre of mass; DRT, dentatorubrothalamic tract; FU, follow-up; POD1, post-operative day 1.

While lesion COM eliminates the lesion size, an accumulated-lesions overlap map was reconstructed to explore the ‘critical lesioning area’ on the normalized DRT coordinate system. The successful treatments accumulated lesions map shows a focused, circular region within the anterolateral DRT quadrat and anteromedial-to-posterolateral smearing, which correlates with the lesions-COMs map (Fig. 8A). Nevertheless, excluding the AM group yields a more focused, circular region with slight anteromedial spreading. Surprisingly, there is a small area (0.375 mm^2^) where all lesions overlap (Fig. 8A). This centre is enclosed by a circular region in which at least 85% of the lesions overlap and is the most strongly correlated with successful and long-lasting outcomes. Its centre is located at (−2.59 mm, 0.65 mm) and has a diameter of 3 mm.

A similar map was generated for the failure treatments. The overlay is more dispersed, with no prominent focus. The maximum value is 76% in a minimal area (0.125 mm^2^), which is not concentric, reflecting a widespread overlay map (Fig. 8B). However, this region partially overlaps with the convergence area of the successful treatments.

The area with the highest difference between the AL-cluster and failure lesions is a circular region of success dominance located in the lateral part of the tract, spreading over both the anterior- and posterior-lateral quarters (Fig. 8C). High values are located laterally in this region and close to the horizontal midline of the tract. The area of maximum difference was defined as a ‘critical area’ and is located in the posterior-lateral margins of the 85% success-overlap circular region. 78% of the success and 19% of the failure lesions cover this area.

### Linear regression results

A linear model was applied to predict the most important parameters for the successful treatment target (COM). The location of the DRT (DRT_X_, DRT_Y_), Ѳ_DRT_ (DRT orientation), and the ratios between the DRT, ML, and PT locations. In addition, a leave-one-out method was applied. The mean distance between the predicted and the lesions COM is 0.51 ± 0.34 mm, with *R^2^* = 0.84, while the leave-one-out 0.86 ± 0.49 mm represents a high-quality, accurate model. Subsequently, this model was applied to the failure treatment group with a mean distance error of 1.61 ± 0.77 mm, which may explain the temporary or partial clinical response.

## Discussion

The challenge of locating the target for tremor control and using tractography as a targeting tool is widely discussed.^10,14,22,25,34,36,47^ Despite its disadvantages, canonical targeting using AC-PC coordinates remains the most common approach.^22^ A personalized, functionally oriented, anatomy-based approach may improve treatment outcomes and reduce side effects. Accurate targeting may result in shorter treatment, reduced number of sonications, and an improved chance of reaching the ablative temperature at the target. In addition, it will improve the patient’s tolerance to the treatment, their experience, and may pave the way for asleep procedures.

Tractography-based targeting methods usually combine the PT, ML, and DRT.^10,22,26,32,34^ In 2011, Coenen *et al*. reported the first successful movement disorder tremor surgery with tractography-based targeting,^30^ and in 2016, Sammartino *et al*. introduced a targeting method based on the PT and ML.^32^ However, almost without exception, tractography-based targeting studies rely on small cohorts and don't encounter long-term outcomes.^10,25,26,32,47^

This study aimed to develop a personalized technique based on functional landmarks to accurately identify the target for tremor treatment using MRgFUS. Using data collected over 10 years at our hospital, we performed personal tractography to delineate the PT, ML, and DRT and retrospectively examined the correlation between lesion characteristics, tracts, and clinical outcomes. Since significant variability exists between patients presenting symptoms, diagnosis, and the longevity of tremor reduction, we mainly focused on the 2-year follow-up results. However, one should consider tremor relapse secondary to disease progression or neuronal plasticity. Excellent tremor control at 3-month follow-up was documented in 76% of the patients and may be considered as optimal targeting. However, we decided to use a longer clinical follow-up to maximize the chance of correlating optimal targeting to long-term tremor reduction (considering a significant reduction in the number of patients).

The lesion's total volume and the area at the ACPC plane showed no significant correlation to the treatment outcome (Fig. 6), suggesting that the location of the lesion is the most critical parameter (rather than its size), as discussed by Buch *et al*.^48^ All anatomical landmarks, such as the AC-PC length and the width of the thalamus (calculated using PT COM), have shown no significant correlation to the outcome (tremor control or QoL improvement). Moreover, the distance between the final lesion location and the original planned target (as standard canonical targeting) was ∼1 mm and didn't correlate with failure or success. Hence, these results emphasize the need for a better targeting approach while utilizing functional imaging to overcome anatomical variabilities.

Our cohort includes PD and ET patients (24, 57, respectively). While all patients underwent the same VIM thalamotomy approach for tremor control, the tremor score (TS) and treated hand tremor score (TS(th)) enable consistent clinical follow-up. Because tracts’ morphology did not show statistically significant differences (Supplementary Table 4), we combined the cohort to identify the tremor-control sweet spot regardless of aetiology. The outcome analysis (Figs 6 and 7) highlighted the correlation to the targeting point with respect to diagnosis. We believe that consistent targeting of tremor control supports the findings and emphasizes the clinical value of the DRT sweet spot.

### Tractography

In this study, we performed personal tractography from scans of 81 ET/PD patients and two different DTI protocols. The tractography analysis relied on anatomical knowledge of the tracts, i.e. anatomical landmarks and trajectories. Furthermore, all tracts were delineated by the same analyst and reviewed with a systematic, uniform tracking approach.

This study provided an opportunity to examine tracts from data acquired in a clinical setting in a personalized manner. While the main characteristics of the tract, such as trajectory, general morphology, and anatomical landmarks, are preserved across patients, no tract is identical to another, and the inter-patient variability of the obtained tracts is noteworthy, particularly for the DRT. Generally, this variability can be characterized by differences in volume, density, fibre count, cortical projections, and morphological sub-features. When applying the same preprocessing and tracking methodology to all patients, the inter-patient variability in tracts may arise from three primary sources: natural variation among patients (clinical and/or anatomical factors), algorithmic differences, and scan artefacts (‘unnatural’ factors).^27^ Tracing and dissection studies can and should serve as the ‘gold standard’ for tractography with respect to the general characteristics of a tract, e.g. anatomical landmarks and course, and should be used to validate clinical data.

We selected the tract COM as the reference point for our analysis. This choice is based on the assumption that the COM is less susceptible to differences in tractography methods, tracking errors, and registration-related interpolation effects than, for example, the perimeter. Choosing different thresholds for tract values will yield a substantially different perimeter. Moreover, evidence suggests that the tract's core is more preserved through different tractography software, even though differences between the tracts exist.^49^ Hence, choosing the COM as a reference point may facilitate the implementation of the presented targeting method using various tractography techniques.

Our results show a linear correlation between the COMs of the PT, ML, and DRT. These correlations underscore the repeatability across patients and support the rationale for using tract COMs. Moreover, it may provide a tract quality evaluation tool that is useful for tract-based targeting.

The DRT orientation (the angle between the *X*-axis and the main DRT diameter—Ѳ_DRT_) varies significantly between patients. However, according to the linear correlations, the wider the thalamus is (greater PT/ML-midline distance), the smaller the Ѳ_DRT_ is (Fig. 3F). Hence, Ѳ_DRT_ can be used as a tool to reflect patient-specific anatomy.

Two DTI protocols were included in this research, differing in the number of diffusion gradient directions (25 and 60). Since the number of diffusion directions may affect the tractography results,^46^ several assessments were made to validate the combination of the two cohorts. Data acquired with 25 directions was more compromised than data acquired with 60 directions, resulting in a higher rate of patients and/or tracts being eliminated from the study. However, there was no significant variability between the protocols in COMs, and the area of the DRT at the ACPC plane (Supplementary Table 2), nor the discussed linear correlation between tracts (Supplementary Table 3). We argue that the analysis of tracts COMs is valid even when using low-quality data for targeting. However, a higher number of diffusion directions are preferable. Further studies across different cohorts should validate the linear correlation between tract COM and COM.

### The role of DRT

A growing body of literature describes the importance of targeting the DRT for tremor reduction using MRgFUS and DBS.^10,14,22,25,26,34^ The DRT contains fibres connecting the cerebellum to the thalamus, reaching the ventralis oralis posterior nucleus (VOP) and the nucleus ventralis intermedius (VIM), which, in turn, project to the primary motor cortex.^30,45^ It comprises decussating and non-decussating regions and regulates motor control.^45,50^ Although there is no standard for using DRT parts in clinical settings, we selected the non-decussating DRT as the primary component for analysis because of its robustness and reliability in patients.

While the DRT area at the ACPC level is 62.68 ± 18.39 mm^2^, we hypothesized that the target should be specified within this area to achieve superior clinical outcomes. Our analysis shows no specific correlation between the percentage of DRT lesioning at the ACPC level and clinical outcomes. Furthermore, because lesions within the DRT lead to unsuccessful treatments, we can infer that overlapping the lesion with the DRT is necessary but insufficient for successful treatment. Analyzing the optimal spot within the DRT by both the COM and cumulative models suggests a specific tract region to be targeted. The medial aspect of the anterior-lateral area of the DRT showed a high concentration of successful treatments. At the same time, failure treatments overlapped with the tract but in different regions (Figs 7 and 8).

Modelling the DRT as an ellipse at the ACPC plane is practical and reliable. However, the DRT orientation inter-patient variability is profound (Mean Ѳ_DRT_ = −25.05°±10.68). Using a normalized model of the DRT may replace other normalized approaches, such as the Montreal Neurological Institute (MNI) coordinate system, and therefore be a more patient-specific approach that saves time and computational resources during analysis. Moreover, using the orientation adds to the model the consideration of a 2D tract rather than a point and may consider homunculus orientation within the DRT fibres.

It should be noted that analyses based on the PT and ML COMs were performed, while no significant correlation with successful treatment was found in either case. However, given the linear regression model (*R*^2^ = 0.84), the correlation between the PT and ML locations and the target should be further investigated.

### ‘Sweet spot’ targeting model

In this study, we presented three novel approaches to define the target for tremor control using MRgFUS. First, model the DRT as an ellipse at the ACPC level. Second, tracts COM (DRT, PT, and ML) are defined as the primary properties that represent the tracts to simplify representation and reduce sensitivity to errors. Third, DRT orientation as a key factor in a linear correlation model to define the target and as a normalized platform for lesion presentation.

While there is no correlation between successful or failed-treatment in the anatomical X-Y coordinate system (AC-PC plane, Fig. 5), well defined clusters were noted for the successful treatments based on the combined model revealed a specific region within DRT-based on both lesions COM and cumulative map (Figs 7 and 8) and in the same time, the failed treatments presented scattered area around it.

Puncturing a specific point within the DRT as a ‘sweet spot’, we analysed successful lesions COM, and recognized two main clusters. The main group, comprising the majority of successful lesions, is located in the anterolateral part of the DRT (AL group). This group may be divided into two clusters: the medial AL (mAL) and the posterolateral AL (plAL). Analyzing lesion COM at 1 year and 2-year follow-up was similar, with consistent results (Fig. 7A and B).

A second small group of successful treatments was observed separately, distant from the main AL group (anteromedial, AM group). No clinical differences or notable anatomical landmarks were noted in those treatments. Several factors may account for this phenomenon: noise and data-processing errors, such as lesion evaluation, COM definition, and registration errors that rely solely on the DRT. Alternatively, inter-patient variability of tremor characteristics or pathophysiology. Thus, it may indicate a different target location, and one can speculate that there is more than one ‘sweet spot’. However, this cluster contains only four treatments hence, it is difficult to define its implications, while further investigation is needed to be unambiguous.

The COM analysis of lesions can be misleading in some cases for three reasons: First, by using the lesion COM, we inevitably assume that all lesions are circular and have the same diameter. Second, it does not account for multiple lesions within a single treatment (in which residual tremor was noted after lesioning and complementary sonications were delivered). Third, by presenting the lesion as a single point, it discards any critical area/volume that must be ablated for a good clinical response.

Hence, we conducted an accumulated-overlap lesion analysis (Fig. 8) to identify the critical region for treatment success, i.e. the area that must be ablated for successful treatment. The accumulated overlap analysis showed results similar to those of the COMs. The medial area of the anterolateral quarter of the DRT showed a high percentage of successful lesions that overlap, creating a focal, circular area (Fig. 8). This region represents the area that should be ablated to achieve a superior outcome. Furthermore, cumulative overlap analysis of failed treatments doesn't present a ‘hot spot’, suggesting the lesion didn't match the ‘critical area’. Further analysis aimed at identifying a region of maximal difference between success- and failure-lesions, accumulated overlap showed a ‘critical area’ with only 19% of the failure-lesions overlapping and 78% of the success-lesions overlapping. This difference map (Fig. 8C), particularly the resulting ‘critical area’, emphasizes the importance of the lesion size, including the posterior-lateral part of the tract, to achieve superior outcomes. The results strengthen the hypothesis that failed treatments do not overlap the ‘sweet spot’ or only partially overlap, implying that the lesion wasn't precise enough, resulting in partial or short-term tremor control.

The linear regression approach showed promising results with *R*^2^ = 0.84. However, since this method is sensitive to outliers, it should be tested on a larger dataset than the two other approaches.

The target defined by the COMs model, based on the mAL cluster, is located at 0.7 mm and 1.06 mm antero-medially relative to the potential targets defined by the AL cluster and the accumulated-overlap approach, respectively. Although the treatments in the plAL group showed profound tremor control and improved QOL, a high profile of SE was observed, suggesting a higher-risk region. A more anteromedial target is considered safer with respect to the internal capsule (which contains the PT) and the ML. Hence, we suggest localizing the treatment target using the COMs approach based on the mAL group while considering a radius of 2.5 mm to include the ‘critical area’ that was found. This region encompasses the area that showed an 87% overlap of success lesions and the potential targets defined using accumulated overlap and the AL cluster (Fig. 8).

In addition, we suggest the AM cluster as an alternative target when insufficient tremor reduction is achieved with the primary target.

### Study limitations

Success/failure criteria aim to classify between treatments. However, QOL is a subjective score and can be influenced by various circumstances that may cause a ‘false negative’, i.e. false failure classification. Patient heterogeneity (ET, PD, MSA, and other diagnoses) may cause classification bias as some patients are rapidly progressive. On the other hand, without a standard of care and drug regimen protocol, some treatments can falsely be classified as success (i.e. good symptom control with medication rather than ablation).

Image analysis may cause significant implant error due to multistep analysis (DTI acquisition, T1-DTI registration, lesion identification, and segmentation). DRT ellipsoid modelling and normalization cause inaccuracies (the effects of personal tractography on the results were discussed above). Echo-planar imaging (EPI) distortion correction couldn’t be applied since the necessary images weren’t acquired. However, a manual review of all registrations by a single rater was performed, and the results’ consistency may support the registration reliability.

Although the cohort size is larger than in similar studies, the number of patients remains a statistical limitation, particularly for binary classification and clustering. In addition, this study may be subject to bias due to its retrospective design, and further prospective testing is warranted.

## Conclusions

In this study, we analyzed pre- and postoperative MRI scans from 81 patients who underwent MRgFUS to evaluate the use of advanced analysis methods as a targeting approach for superior clinical outcomes. Using tractography, we delineated three tracts for each patient. After performing registration, lesion segmentation, and geometric measurements, we modelled the DRT and statistically analyzed the results.

We propose an innovative, personalized method for detecting the target location for tremor reduction via MRgFUS. This technique tailors the target to the patient based on functional landmarks. We establish personal tractography as a tool for personal thalamus navigation and assess the significance of targeting a specific area of the DRT for treatment outcome, which may contribute to understanding tremor pathophysiology. A prospective study should be conducted to validate and demonstrate the superiority of the proposed targeting approach over the conventional method.

## Supplementary Material

fcag170_Supplementary_Data

## Data Availability

The data that support the findings of this study are available from the corresponding author, upon reasonable request. Scripts generated for the purpose of statistical analyses are included in the Supplementary material.

## References

[1] Lorenz D, Schwieger D, Moises H, Deuschl G. Quality of life and personality in essential tremor patients. Mov Disord. 2006;21(8):1114–1118. 16622851 10.1002/mds.20884

[2] Louis ED, Machado DG. Tremor-related quality of life: A comparison of essential tremor vs. Parkinson’s disease patients. Parkinsonism Relat Disord. 2015;21(7):729–735. 25952960 10.1016/j.parkreldis.2015.04.019 PMC4764063

[3] Zaaroor M, Sinai A, Goldsher D, Eran A, Nassar M, Schlesinger I. Magnetic resonance–guided focused ultrasound thalamotomy for tremor: A report of 30 Parkinson’s disease and essential tremor cases. J Neurosurg. 2018;128(1):202–210. 28298022 10.3171/2016.10.JNS16758

[4] Elias WJ, Huss D, Voss T, et al A pilot study of focused ultrasound thalamotomy for essential tremor. N Engl J Med. 2013;369(7):640–648. 23944301 10.1056/NEJMoa1300962

[5] Lev-Tov L, Barbosa DAN, Ghanouni P, Halpern CH, Buch VP. Focused ultrasound for functional neurosurgery. J Neurooncol. 2021;156:17–22. 34383232 10.1007/s11060-021-03818-3

[6] Benabid AL, Pollak P, Louveau A, Henry S, de Rougemont J. Combined (Thalamotomy and Stimulation) stereotactic surgery of the VIM thalamic nucleus for bilateral Parkinson disease. Stereotact Funct Neurosurg. 1987;50(1–6):344–346. 10.1159/000100803 3329873

[7] Elias WJ, Lipsman N, Ondo WG, et al A randomized trial of focused ultrasound thalamotomy for essential tremor. N Engl J Med. 2016;375(8):730–739. 27557301 10.1056/NEJMoa1600159

[8] Saluja S, Barbosa DAN, Parker JJ, et al Case report on deep brain stimulation rescue after suboptimal MR-guided focused ultrasound thalamotomy for essential tremor: A tractography-based investigation. Front Hum Neurosci. 2020;14:191. 32676015 10.3389/fnhum.2020.00191 PMC7333679

[9] Tasker RR, Siqueira J, Hawrylyshyn P, Organ LW. What happened to VIM thalamotomy for Parkinson’s disease? Stereotact Funct Neurosurg. 1983;46(1–4):68–83. 10.1159/000101245 6367656

[10] Bruno F, Catalucci A, Varrassi M, et al Comparative evaluation of tractography-based direct targeting and atlas-based indirect targeting of the ventral intermediate (Vim) nucleus in MRgFUS thalamotomy. Sci Rep. 2021;11(1):13538. 34188190 10.1038/s41598-021-93058-2 PMC8241849

[11] Hirai T, Ohye C, Nagaseki Y, Matsumura M. Cytometric analysis of the thalamic ventralis intermedius nucleus in humans. J Neurophysiol. 1989;61(3):478–487. 2709094 10.1152/jn.1989.61.3.478

[12] Morel A, Magnin M, Jeanmonod D. Multiarchitectonic and stereotactic atlas of the human thalamus. J Comp Neurol. 1997;387(4):588–630. doi: 10.1002/(sici)1096-9861(19971103)387:4 9373015

[13] Najdenovska E, Tuleasca C, Jorge J, et al Comparison of MRI-based automated segmentation methods and functional neurosurgery targeting with direct visualization of the ventro-intermediate thalamic nucleus at 7T. Sci Rep. 2019;9(1):1119. 30718634 10.1038/s41598-018-37825-8 PMC6361927

[14] Gravbrot N, Saranathan M, Pouratian N, Kasoff WS. Advanced imaging and direct targeting of the motor thalamus and dentato-rubro-thalamic tract for tremor: A systematic review. Stereotact Funct Neurosurg. 2020;98(4):220–240. 32403112 10.1159/000507030

[15] Su JH, Thomas FT, Kasoff WS, et al Thalamus optimized multi atlas segmentation (THOMAS): Fast, fully automated segmentation of thalamic nuclei from structural MRI. NeuroImage. 2019;194:272–282. 30894331 10.1016/j.neuroimage.2019.03.021 PMC6536348

[16] Calabrese E . Diffusion tractography in deep brain stimulation surgery: A review. Front Neuroanat. 2016;10:45. 27199677 10.3389/fnana.2016.00045 PMC4852260

[17] Brierley JB, Beck E. The significance in human stereotactic brain surgery of individual variation in the diencephalon and globus pallidus. J Neurol Neurosurg Psychiatry. 1959;22(4):287–298. 13804402 10.1136/jnnp.22.4.287 PMC497393

[18] Nowinski WL . Anatomical targeting in functional neurosurgery by the simultaneous use of multiple schaltenbrand-wahren brain atlas microseries. Stereotact Funct Neurosurg. 1998;71(3):103–116. 10420144 10.1159/000029654

[19] Chen T, Mirzadeh Z, Chapple KM, et al Intraoperative test stimulation versus stereotactic accuracy as a surgical end point: A comparison of essential tremor outcomes after ventral intermediate nucleus deep brain stimulation. J Neurosurg. 2018;129(2):290–298. 29027853 10.3171/2017.3.JNS162487

[20] Burchiel KJ, McCartney S, Lee A, Raslan AM. Accuracy of deep brain stimulation electrode placement using intraoperative computed tomography without microelectrode recording: Clinical article. J Nutr Sci. 2013;119(2):301–306. 10.3171/2013.4.JNS122324 23724986

[21] Vassal F, Coste J, Derost P, et al Direct stereotactic targeting of the ventrointermediate nucleus of the thalamus based on anatomic 1.5-T MRI mapping with a white matter attenuated inversion recovery (WAIR) sequence. Brain Stimul. 2012;5(4):625–633. 22405744 10.1016/j.brs.2011.10.007

[22] Lehman VT, Lee KH, Klassen BT, et al MRI and tractography techniques to localize the ventral intermediate nucleus and dentatorubrothalamic tract for deep brain stimulation and MR-guided focused ultrasound: A narrative review and update. Neurosurg Focus. 2020;49(1):E8. 10.3171/2020.4.FOCUS20170 PMC8032505 32610293

[23] Deistung A, Schäfer A, Schweser F, Biedermann U, Turner R, Reichenbach JR. Toward in vivo histology: A comparison of quantitative susceptibility mapping (QSM) with magnitude-, phase-, and R2⁎-imaging at ultra-high magnetic field strength. NeuroImage. 2013;65:299–314. 23036448 10.1016/j.neuroimage.2012.09.055

[24] Sudhyadhom A, Haq IU, Foote KD, Okun MS, Bova FJ. A high resolution and high contrast MRI for differentiation of subcortical structures for DBS targeting: The fast gray matter acquisition T1 inversion recovery (FGATIR). NeuroImage. 2009;47:T44–T52. 19362595 10.1016/j.neuroimage.2009.04.018

[25] Morishita T, Higuchi MA, Kobayashi H, Abe H, Higashi T, Inoue T. A retrospective evaluation of thalamic targeting for tremor deep brain stimulation using high-resolution anatomical imaging with supplementary fiber tractography. J Neurol Sci. 2019;398:148–156. 30716581 10.1016/j.jns.2019.01.025

[26] Chazen JL, Sarva H, Stieg PE, et al Clinical improvement associated with targeted interruption of the cerebellothalamic tract following MR-guided focused ultrasound for essential tremor. J Neurosurg. 2018;129(2):315–323. 29053074 10.3171/2017.4.JNS162803

[27] Behrens TEJ, Sotiropoulos SN, Jbabdi S. MR diffusion tractography. In: Diffusion MRI. Elsevier; 2014:429–451.

[28] Mori S, van Zijl PCM. Fiber tracking: Principles and strategies—A technical review. NMR Biomed. 2002;15(7–8):468–480. 12489096 10.1002/nbm.781

[29] See AAQ, King NKK. Improving surgical outcome using diffusion tensor imaging techniques in deep brain stimulation. Front Surg. 2017;4:54. 29034243 10.3389/fsurg.2017.00054 PMC5625016

[30] Coenen VA, Allert N, Mädler B. A role of diffusion tensor imaging fiber tracking in deep brain stimulation surgery: DBS of the dentato-rubro-thalamic tract (drt) for the treatment of therapy-refractory tremor. Acta Neurochir (Wien). 2011;153(8):1579–1585. 21553318 10.1007/s00701-011-1036-z

[31] Fenoy AJ, Schiess MC. Deep brain stimulation of the dentato-rubro-thalamic tract: Outcomes of direct targeting for tremor. Neuromodulation. 2017;20(5):429–436. 28256785 10.1111/ner.12585

[32] Sammartino F, Krishna V, King NKK, et al Tractography-based ventral intermediate nucleus targeting: Novel methodology and intraoperative validation. Mov Disord. 2016;31(8):1217–1225. 27214406 10.1002/mds.26633 PMC5089633

[33] Anthofer J, Steib K, Fellner C, Lange M, Brawanski A, Schlaier J. The variability of atlas-based targets in relation to surrounding major fibre tracts in thalamic deep brain stimulation. Acta Neurochir (Wien). 2014;156(8):1497–1504. 24829155 10.1007/s00701-014-2103-z

[34] Coenen VA, Allert N, Paus S, Kronenbürger M, Urbach H, Mädler B. Modulation of the cerebello-thalamo-cortical network in thalamic deep brain stimulation for tremor. Neurosurgery. 2014;75(6):657–670. 25161000 10.1227/NEU.0000000000000540

[35] Miller TR, Zhuo J, Eisenberg HM, et al Targeting of the dentato-rubro-thalamic tract for MR-guided focused ultrasound treatment of essential tremor. Neuroradiol J. 2019;32(6):401–407. 31407957 10.1177/1971400919870180 PMC6856993

[36] Ranjan M, Elias GJB, Boutet A, et al Tractography-based targeting of the ventral intermediate nucleus: Accuracy and clinical utility in MRgFUS thalamotomy. J Neurosurg. 2020;133(4):1002–1009. 31561221 10.3171/2019.6.JNS19612

[37] Yamada K, Akazawa K, Yuen S, et al MR imaging of ventral thalamic nuclei. AJNR Am J Neuroradiol. 2010;31(4):732–735. 19926703 10.3174/ajnr.A1870 PMC7964215

[38] Sinai A, Nassar M, Sprecher E, Constantinescu M, Zaaroor M, Schlesinger I. Focused ultrasound thalamotomy in tremor dominant Parkinson’s disease: Long-term results. JPD. 2022;12(1):199–206. 34602500 10.3233/JPD-212810 PMC8842770

[39] Leemans A, Jeurissen B, Sijbers J, Jones DK. ExploreDTI: a graphical toolbox for processing, analyzing, and visualizing diffusion MR data. In: *17th Annual Meeting of Intl Soc Mag Reson Med*. Hawaii, USA. 2009:3537.

[40] Smith SM . Fast robust automated brain extraction. Hum Brain Mapp. 2002;17(3):143–155. 12391568 10.1002/hbm.10062 PMC6871816

[41] Jenkinson M, Beckmann CF, Behrens TEJ, Woolrich MW, Smith SM. FSL. NeuroImage. 2012;62(2):782–790. 21979382 10.1016/j.neuroimage.2011.09.015

[42] Smith SM, Jenkinson M, Woolrich MW, et al Advances in functional and structural MR image analysis and implementation as FSL. NeuroImage. 2004;23:S208–S219. 15501092 10.1016/j.neuroimage.2004.07.051

[43] Woolrich MW, Jbabdi S, Patenaude B, et al Bayesian analysis of neuroimaging data in FSL. NeuroImage. 2009;45(1):S173–S186. 19059349 10.1016/j.neuroimage.2008.10.055

[44] Tustison NJ, Cook PA, Holbrook AJ, *et al*. The ANTsX ecosystem for quantitative biological and medical imaging. *Sci Rep*. 2021;11(1):9068. doi:10.1038/s41598-021-87564-6. PMC8079440 33907199

[45] Petersen KJ, Reid JA, Chakravorti S, et al Structural and functional connectivity of the nondecussating dentato-rubro-thalamic tract. NeuroImage. 2018;176:364–371. 29733955 10.1016/j.neuroimage.2018.04.074 PMC6002752

[46] Callaghan F, Maller JJ, Welton T, Middione MJ, Shankaranarayanan A, Grieve SM. Toward personalised diffusion MRI in psychiatry: Improved delineation of fibre bundles with the highest-ever angular resolution in vivo tractography. Transl Psychiatry. 2018;8(1):91. 29691374 10.1038/s41398-018-0140-8 PMC5915595

[47] Tian Q, Wintermark M, Jeffrey Elias W, et al Diffusion MRI tractography for improved transcranial MRI-guided focused ultrasound thalamotomy targeting for essential tremor. Neuroimage Clin. 2018;19:572–580. 29984165 10.1016/j.nicl.2018.05.010 PMC6029558

[48] Buch VP, Purger D, Datta A, et al “Quality over quantity:” smaller, targeted lesions optimize quality of life outcomes after MR-guided focused ultrasound thalamotomy for essential tremor. Front Neurol. 2024;15:1450699. 39610701 10.3389/fneur.2024.1450699 PMC11603361

[49] Christidi F, Karavasilis E, Samiotis K, Bisdas S, Papanikolaou N. Fiber tracking: A qualitative and quantitative comparison between four different software tools on the reconstruction of major white matter tracts. Eur J Radiol Open. 2016;3:153–161. 27489869 10.1016/j.ejro.2016.06.002 PMC4959946

[50] Meola A, Comert A, Yeh FC, Sivakanthan S, Fernandez-Miranda JC. The nondecussating pathway of the dentatorubrothalamic tract in humans: Human connectome-based tractographic study and microdissection validation. J Nutr Sci. 2016;124(5):1406–1412. 10.3171/2015.4.JNS142741 26452117

