# Supplementary material for: Personalized tremor control targeting for MR-guided focused ultrasound using tractography
Source: Brain Commun. 2026 Jun 22;8(3):fcag170. doi: 10.1093/braincomms/fcag170 (PMC13284421; doi:10.1093/braincomms/fcag170)
Supplement: fcag170_Supplementary_Data [file fcag170_supplementary_data.zip › Supplementary_material.docx]

**Supplementary Data**

1. **Tracking and ROIs**

The software ExploreDTI^1^ was used for tractography analysis, applying distortions correction and the constrained spherical deconvolution method.

Our tract reconstruction approach included two steps. First, the relevant ROIs were marked to identify potential fibers of the tract. Second, the obtained fibers were validated against a priori anatomical knowledge, and irrelevant fibers were excluded to yield reliable tracts that corresponded to anatomical and histological data. Note that all tracts were reconstructed by the same author (NS) and validated across all patients.

The most relevant tracts targeting the VIM are the DRT, ML, and PT, which help to identify the patient-specific functional anatomy of the VIM, the tremor pathway, and its surroundings. In general, our approach was to broaden the tracking limits defined by the tracking parameters (i.e., increase the possibility for false-positive fibers and decrease false-negative fibers) and implement manual tractography analysis utilizing a priori anatomical knowledge to avoid loss of fibers, especially if the scan is compromised or has algorithm errors. Each tract was classified as good/descent/poor tract based on fiber density, anatomical characterization, and morphology.

Tractography studies for tremor reduction using DBS or MRgFUS present three main approaches: DRT-only, ML-PT, or all three tracts tracking.^2–6^ Delineating all three tracts is beneficial for verification, particularly the boundaries, and for increasing confidence and assessing the tracts' quality.^2,7^ For each tract, regions of interest (ROIs) were marked using the fractional anisotropy map for tracing. There is no "gold standard" of ROIs localization for DRT tracking. Combinations of the red nucleus, superior cerebellar peduncle, dentate nucleus, and primary motor cortex are commonly used.^3,4,6,8^ For the DRT, ROIs were placed over the red nucleus (RN) region and the ipsilateral primary motor cortex. At the same time, only fibers projecting to the cerebellum through the superior cerebellar peduncle were kept.^3,8^ Initially, we chose to use an ROI on the RN to track decussating and non-decussating fibers; however, in the majority of the scans, the decussating tract wasn't observed.^9^ Thus, we based our analysis on the non-decussating fibers. The PT was delineated by ROIs in the cerebral peduncle and the ipsilateral primary motor cortex. The dorsal column at the brain stem and the ipsilateral primary sensory cortex were chosen as ROIs for the ML.^5^

Subsequently, all the tracts were reviewed for quality assessment to validate anatomical characterization and ensure uniform cleaning. Tracts that were highly sparse, had exceptionally low volume, or were extremely diverse from typical tract morphology were classified as poor quality and eliminated. In addition, patients with two or more tracts of significantly poor quality were excluded from the study.

1. **25 Vs 60 Directions DTI analysis**

Acquisition of DTI requires a minimum of six non-collinear gradient directions to capture the vectorial nature of water diffusion in three-dimensional space for fibers reconstructions. Increasing the number of directions is essential for improving the signal-to-noise ratio (SNR) and the accuracy of derived metrics like Fractional Anisotropy (FA). More importantly, higher directional resolution is critical for resolving complex white matter architectures, such as "crossing fibers," and for performing robust fiber tractography. In our center, in the first 6 years, DTI was acquired with 25 directions, and after software update, with 60 directions. We analyzed the data retrospectively and found no statistically significant difference (Table S2) between protocols (i.e., 25 directions are sufficient to create reliable tracts) and between linear correlations across tracts (Table S3). Hence, we conclude that the data can be combined for “sweet-spot” targeting analysis.

**Supplementary Table 1: Presenting Lesion geometrical evaluation with good tremor control (more than 60% TR) at 12 months and 24 months FU, mean ± STD [min, max]**

|  | **12 Months** | **24 Months** |
| --- | --- | --- |
| Lesion Area (AC-PC) [mm^2^] | 25.98±8.5 [6.65, 50.96] | 26.64±8.7 [6.65, 50.96] |
| % lesioned DRT area (AC-PC plane) | 27.31±11.1 [8.04, 54.93] | 29.44±11.96 [8.04, 54.93] |
| overlap area DRT-lesion (AC-PC) [mm^2^] | 16.95±5.73 [5.17, 28.8] | 18.35±6.53 [5.17, 33.97] |
| Lesion Volume [mm^3^] | 177.02±69.06 [34.12, 334.13] | 176.94±68.94 [34.12, 334.13] |
| Lesion Volume under the AC-PC [mm^3^] | 70.17±35.78 [7.98, 144.69] | 70.13±35.93 [7.98, 144.69] |
| % lesioned DRT volume | 21.16±8.03 [6.7, 40.23] | 22.1±8.11 [6.7, 40.23] |

FU = follow-up, TR = tremor reduction, DRT = dentatorubrothalamic tract, AC = anterior commissure, PC = posterior commissure, STD = standard deviation.

**Supplementary Table 2: Presenting DRT morphology across the two acquisition protocols, mean ± STD**

|  | **25 directions** | **60 directions** | **p-value** |
| --- | --- | --- | --- |
| DRT area at AC-PC plane | 60.5±16.79 | 70.73±21.52 | 0.15 |
| DRT orientation at AC-PC plane | -24.79±10.03 | -26.01±12.71 | 0.76 |
| D1 | 15.04±2.54 | 15.03±2.61 | 0.98 |
| D2 | 5.91±1.54 | 6.78±1.36 | 0.07 |
| Number of fibers | 745.42±480.82 | 928.69±700.31 | 0.41 |

DRT = dentatorubrothalamic tract, AC = anterior commissure, PC = posterior commissure, D1 = major ellipse diameter, D2 = minor ellipse diameter, STD = standard deviation.

**Supplementary Table 3: Presenting the distance from the regression line (fitted to the data acquired with 25 directions), mean ± STD**

| **Linear regression (x, y)** | **25 directions (mm)** | **60 directions (mm)** | **p-value** |
| --- | --- | --- | --- |
| ML_X_, DRT_X_ | 0.73±0.59 | 1.18±0.79 | 0.083 |
| ML_X_, PT_X_ | 0.65±0.5 | 0.57±0.54 | 0.658 |
| PT_X_, DRT_X_ | 0.73±0.71 | 1.08±0.79 | 0.175 |
| ML_Y_, PT_Y_ | 0.66±0.52 | 0.51±0.53 | 0.382 |
| AC-PC, PT_X_ | 1.12±0.75 | 1.16±1.05 | 0.921 |
| ML_X_, DRT-orientation | 4.62±3.72 | 6.15±3.25 | 0.176 |

PT = pyramidal tract, ML = medial lemniscus, DRT = dentatorubrothalamic tract, AC = anterior commissure, PC = posterior commissure, STD = standard deviation.

**Supplementary Table 4: Presenting DRT morphology across ET and PD patients, mean ± STD [min, max]**

|  | **ET** | **PD** | **p-value** |
| --- | --- | --- | --- |
| DRT area at AC-PC plane | 64.22±19.84 | 59.64±12.91 | 0.37 |
| DRT orientation at AC-PC plane | -23.37±9.74 | -30.7±9.61 | 0.04 |
| D1 | 15.41±2.57 | 14.08±1.95 | 0.08 |
| D2 | 6.13±1.64 | 6.18±1.4 | 0.91 |
| Number of fibers | 863.81±558.34 | 602.5±476.14 | 0.14 |

ET = essential tremor, PD = Parkinson’s disease, DRT = dentatorubrothalamic tract, AC = anterior commissure, PC = posterior commissure, STD = standard deviation.

**Supplementary References**

1. Leemans A, Jeurissen B, Sijbers J, Jones DK. ExploreDTI: a graphical toolbox for processing, analyzing, and visualizing diffusion MR data. 1.

2. Lehman VT, Lee KH, Klassen BT, et al. MRI and tractography techniques to localize the ventral intermediate nucleus and dentatorubrothalamic tract for deep brain stimulation and MR-guided focused ultrasound: a narrative review and update. Neurosurg Focus. 2020;49(1):E8. doi:10.3171/2020.4.FOCUS20170

3. Coenen VA, Allert N, Mädler B. A role of diffusion tensor imaging fiber tracking in deep brain stimulation surgery: DBS of the dentato-rubro-thalamic tract (drt) for the treatment of therapy-refractory tremor. Acta Neurochir (Wien). 2011;153(8):1579-1585. doi:10.1007/s00701-011-1036-z

4. Fenoy AJ, Schiess MC. Deep Brain Stimulation of the Dentato-Rubro-Thalamic Tract: Outcomes of Direct Targeting for Tremor. Neuromodulation Technol Neural Interface. 2017;20(5):429-436. doi:10.1111/ner.12585

5. Sammartino F, Krishna V, King NKK, et al. Tractography‐Based Ventral Intermediate Nucleus Targeting: Novel Methodology and Intraoperative Validation. Mov Disord. 2016;31(8):1217-1225. doi:10.1002/mds.26633

6. Miller TR, Zhuo J, Eisenberg HM, et al. Targeting of the dentato-rubro-thalamic tract for MR-guided focused ultrasound treatment of essential tremor. Neuroradiol J. 2019;32(6):401-407. doi:10.1177/1971400919870180

7. Boutet A, Ranjan M, Zhong J, et al. Focused ultrasound thalamotomy location determines clinical benefits in patients with essential tremor. Brain. 2018;141(12):3405-3414. doi:10.1093/brain/awy278

8. Meola A, Comert A, Yeh FC, Sivakanthan S, Fernandez-Miranda JC. The nondecussating pathway of the dentatorubrothalamic tract in humans: human connectome-based tractographic study and microdissection validation. J Neurosurg. 2016;124(5):1406-1412. doi:10.3171/2015.4.JNS142741

9. Coenen VA, Allert N, Paus S, Kronenbu¨rger M, Urbach H, Ma¨dler B. Modulation of the Cerebello-Thalamo-Cortical Network in Thalamic Deep Brain Stimulation for Tremor. Neurosurgery. 2014;75(6):657-670. doi:10.1227/NEU.0000000000000540
